# Enhancing Quality of Life for Individuals with Stroke (EQL): a study protocol for co-creating a social support and context-informed intervention to improve self-management, health and well-being in older adults recovering at home

**DOI:** 10.1136/bmjopen-2025-110976

**Published:** 2026-04-24

**Authors:** Maya Kylén, Marcus Falk Johansson, Linnea McCarthy, Louise Meijering, Signe Tomsone, Malin Tistad, Marie Elf

**Affiliations:** 1 Faculty of Health Science, Kristianstad University, Kristianstad, Sweden; 2 Dalarna University School of Health and Welfare, Falun, Dalarna County, Sweden; 3 Department of Health Sciences, Lund University, Lund, Sweden; 4 Population Research Centre, Urban and Regional Studies Institute, University of Groningen Faculty of Spatial Sciences, Groningen, The Netherlands; 5 Department of Rehabilitation, Riga Stradins University, Riga, Latvia

**Keywords:** Self-Management, Implementation Science, Stroke, REHABILITATION MEDICINE, Hospital to Home Transition

## Abstract

**Introduction:**

Enhancing Quality of Life for Individuals with Stroke (EQL-stroke) is an international, collaborative multiphase project aiming to strengthen supported self-management for older adults recovering from stroke at home in Sweden, Latvia and the Netherlands. Existing poststroke pathways may provide insufficient support for self-management during the transition from hospital to home, and there is limited evidence on interventions that integrate social networks and everyday environmental context.

**Methods and analysis:**

EQL-stroke uses a participatory, multimethod design across three phases. Phase I generates knowledge through policy review, qualitative interviews and people–place mapping (~25 participants per country) and includes cross-cultural adaptation of the Collective Efficacy of Networks Scale. Phase II co-designs and specifies a tailored social network-informed supported self-management intervention (the Network-Based Intervention), including core components and principles for local adaptation (~15 participants per country). Phase III will recruit approximately 20–40 stroke survivors for a single-arm pilot feasibility study with an 8-week follow-up and embedded process evaluation to assess feasibility, acceptability and fidelity in routine practice.

**Ethics and dissemination:**

Ethical approval has been obtained from the Swedish Ethical Review Authority (reg. no. 2025-00083-01), the Rīgas Stradiņa Universitāte Research Ethical Committee (reg. no. Rīgas Stradiņa Universitāte Research Ethical Committee) and the Research Ethics Committee of the Faculty of Spatial Sciences, University of Groningen (reg. no. 2025-07). Findings will be disseminated through peer-reviewed publications, stakeholder engagement activities and patient/public channels.

**STRENGTHS AND LIMITATIONS OF THIS STUDY**

- This multiphase protocol integrates policy review, qualitative inquiry, co-design and pilot feasibility testing within a structured development framework.
- The intervention is developed using participatory co-design across three different European healthcare systems.
- A pragmatic single-arm pilot feasibility design allows assessment of recruitment, retention, fidelity and acceptability in routine practice.
- Inclusion of older adults with mild to moderate impairments may limit applicability to those with more severe stroke-related disabilities.
- Cultural and healthcare system differences may affect implementation and transferability across contexts.

## Background

 This protocol outlines Enhancing Quality of Life for Individuals with Stroke (EQL), an international collaborative multiphase research project aiming to improve the situation of older adults recovering from a stroke at home, funded through the Transforming Health and Care Systems (THCS) initiative.^1^ EQL-stroke will co-create a tailored supported self-management intervention that integrates social networks and everyday environmental context (the Network-Based Intervention (NBI)). The NBI is designed to strengthen self-management after stroke by mobilising the survivor’s social network and attending to the contexts and environments of daily life. The protocol comprises three clearly defined phases: phase I, knowledge generation; phase II, co-design and intervention specification; and phase III, pragmatic pilot feasibility testing (pre–post mixed methods, including an 8-week follow-up and process evaluation).

EQL brings together Sweden, Latvia and the Netherlands to address limitations in poststroke rehabilitation during the transition from hospital to home, where support for self-management may be insufficient. This is increasingly relevant as care and rehabilitation take place at home and in the community.^2^,^4^ The European Stroke Organization (ESO)^5^ and the Stroke Association for Europe (SAFE)^6^ priorities for life after stroke highlight gaps in longer-term support, including supported self-management, social support and attention to everyday environmental context in rehabilitation. EQL-stroke addresses these gaps by co-designing an intervention that mobilises social networks and attends to everyday context, and by evaluating feasibility and acceptability of delivery in routine practice across different healthcare systems. The project is participatory and co-creative, involving people with lived experience of stroke, family members and healthcare professionals, and will specify the intervention and its adaptation principles for use across contexts.

The THCS framework^7^ calls for integrated, person-centred solutions that are responsive to variation across European health systems and address inequities in care. EQL-stroke aligns with these priorities by focusing on older adults recovering from stroke at home and strengthening supported self-management through social networks and everyday environmental context, dimensions that are often underdeveloped in poststroke care pathways.

EQL-stroke will contribute (1) a cross-country synthesis of how self-management support is addressed in policy and practice; (2) empirical insights into how social networks and everyday environments shape self-management after stroke; (3) a clearly specified supported self-management intervention with adaptation principles and (4) a cross-culturally adapted measure of network efficacy for future evaluation.

### Stroke incidence, unmet needs and current rehabilitation practices

Stroke is a leading cause of long-term disability worldwide and is often associated with reduced quality of life.^8^ Survivors frequently experience persistent physical, cognitive and emotional impairments,^9^ which may limit their ability to engage in meaningful activities and participate fully in everyday life.^10 11^ These restrictions can contribute to social isolation,^12^ decreased quality of life and an increased risk of adverse events such as falls. As populations age and acute stroke treatment improves, more people live with long-term rehabilitation and support needs, increasing demands on health and welfare systems in Europe.^5 13 14^ Annual stroke incidence remains substantial in the participating countries (eg, Sweden, Latvia and the Netherlands).^6^ Although mortality has declined, stroke burden and care needs remain high. Stroke-related costs are projected to increase by 44% by 2040. However, the quality and availability of stroke care in Europe remain inconsistent.^5 14^ Many people with stroke report notable unmet needs, such as insufficient access to healthcare, constrained social support, struggles in their efforts to manage their daily activities and a lack of tailored rehabilitation.^14^,^17^ Older adults have much worse access to rehabilitation after suffering stroke, even though they may exhibit severe disabilities.^18^ This situation highlights the need for interventions that can improve life after stroke and better support recovery at home.

EQL-stroke brings together Sweden, Latvia and the Netherlands, three countries with different healthcare systems and cultural contexts. Sweden provides a strong welfare-based system but faces rural–urban inequities in access to stroke rehabilitation.^5 14 19 20^ Latvia represents settings with more resource-constrained and uneven access to specialised rehabilitation. The Netherlands offers advanced community and integrated care models within a semiprivatised healthcare system.^5^ Given that all these countries are characterised by inequalities in stroke care,^5 21^ this cross-country design supports development of an intervention that can be adapted to different healthcare systems while remaining responsive to the needs of diverse populations.

Self-management support is a promising approach for improving health outcomes^22 23^ and is particularly relevant for individuals living with long-term conditions such as stroke.^24^ It can strengthen knowledge, skills and conﬁdence to manage their health and everyday lives, thereby improving their quality of life.^24^ Nevertheless, existing interventions and support often focus primarily on disease control and may insufficiently address factors that impact self-management such as social support, health literacy, ethnicity, culture and the everyday environment where recovery takes place.^12^

Research highlights that social support and supportive environments are important for stroke rehabilitation and well-being.^1525^,^27^ Across long-term conditions, social networks can support health behaviours, access to health resources and health outcomes.^28 29^ Additionally, collective eﬃcacy, the belief that a group can achieve common goals, may complement individual self-efficacy.^30^ However, the specific role of social networks and collective efficacy in supported self-management after stroke remains underexplored.^31^ Addressing this gap is vital, especially as more rehabilitation and follow-up take place at home, where everyday places and relationships may influence recovery.

Our previous research and related studies show that the home and local environment significantly impact recovery and participation but are not yet fully integrated into the rehabilitation.^2732^,^38^ Many people often report problems adapting to home and local environments post stroke, including accessibility barriers, reduced participation in routine and meaningful activities, and challenges maintaining social relationships.^32^,^37^ These findings align with other studies indicating that environmental factors can affect daily participation at both individual and societal levels for people with physical disabilities, including those resulting from stroke.^38^ These findings point to a need for feasible ways to identify and understand the social and spatial networks that support recovery, to inform targeted, person-centred interventions.

In stroke rehabilitation, previously studied self-management interventions have commonly included components such as education/information provision, problem-solving, goal setting and behaviour change support, often delivered by healthcare professionals within rehabilitation services.^39 40^ However, fewer interventions have explicitly treated the person’s wider social network and everyday environmental context as core components of supported self-management in home-based recovery.^27 31^

Evidence on poststroke self-management support remains limited, especially for interventions that can be adapted across healthcare contexts. EQL-stroke will co-create and feasibility-test a social network-informed intervention in routine practice, aligning with European calls for stronger long-term, person-centred stroke support and reduced inequities (WHO,^16^ ESO^5^ and SAFE^6^).

### Aim and objectives

The overall aim of EQL-stroke is to improve rehabilitation experiences, health and well-being among older adults recovering from stroke at home in Sweden, Latvia and the Netherlands. To achieve this, the project will develop and primarily evaluate the feasibility of a supported self-management intervention that integrates social networks and everyday environmental context.

To achieve this aim we will

1. Describe how self-management support is currently addressed in policy and practice in each participating country and identify gaps relevant to home-based recovery.
2. Generate empirical knowledge about how social networks (and the support they provide) and everyday environmental context shape self-management after stroke, drawing on interviews and people–place mapping with stroke survivors, family members and professionals.
3. Co-design and specify the intervention, including its core components, intended functions and principles for local adaptation.
4. Assess the feasibility, acceptability and fidelity of delivering the intervention in routine practice through a pragmatic pilot feasibility study (single-arm pre–post mixed methods with an 8-week follow-up), and to refine procedures and candidate outcome measures for future evaluation.
5. Cross-culturally adapt and assess the content validity and usability of a measure of network efficacy (Collective Efficacy Network Scale (CENS) for use across participating contexts.

### Theoretical framework

EQL is grounded in a person-centred approach,^16^ with a focus on supported self-management, social support and everyday environments. Social cognitive theory^41^ provides the overarching behavioural framework, emphasising the reciprocal interactions between personal, behavioural and environmental factors. Within this framework, collective efficacy, defined as a group’s shared belief in its ability to achieve common goals, is treated as a key mechanism through which support from social networks may influence self-management and well-being.^30 41^ In addition, we adopt a salutogenic perspective,^42^ focusing on the promotion of well-being and acknowledging the crucial roles of social and environmental determinants in shaping health outcomes.^43 44^

To understand home-based recovery after stroke, EQL draws on person‒environment interactions, including the ecological model of ageing,^45^ the person–environment–occupation model^46^ and the International Classification of Functioning, Disability and Health.^47^ These perspectives inform our analysis of how environmental factors such as accessibility and resources interact with individual capacities and daily activities. A social ecological perspective is used to conceptualise the person’s social network and the multilevel influences on self-management and participation.^48 49^

The life–space mobility framework contributes with an understanding of everyday movement, place use and social participation.^50^ EQL is informed by intersectionality,^51^ which considers various intersecting factors, such as gender, ethnicity, income and geography, to address structural inequities. To develop the intervention, we use the framework posited by the Medical Research Council (MRC)^51^ and value-sensitive design^52^ to meet diverse stakeholders’ needs and values.

## Methods and analysis

### Overview of the study design

**Figure 1:**
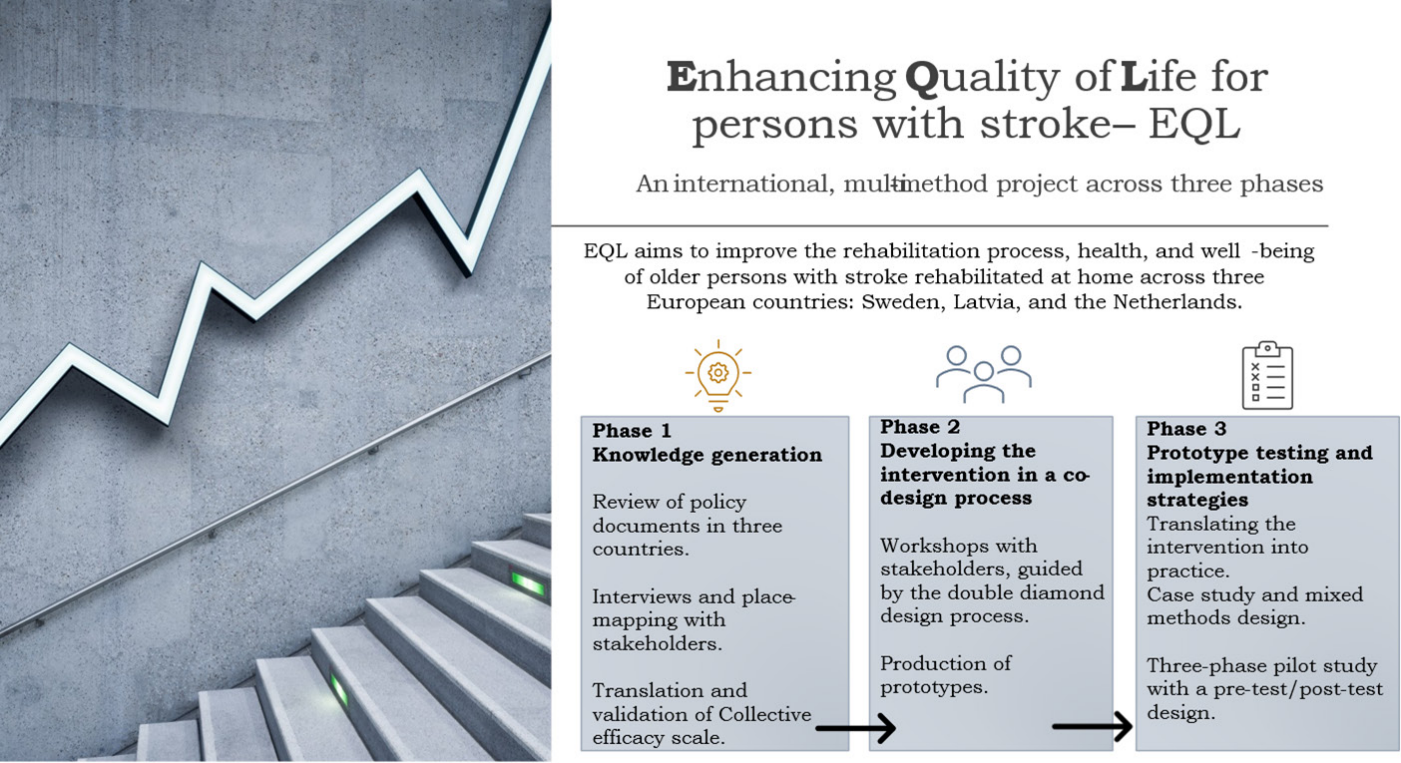
Overview of Enhancing Quality of Life for Individuals with Stroke (EQL) study design.

EQL-stroke is a multiphase, international, multimethod study (2024–2027) organised into three phases (figure 1) and conducted in Sweden, Latvia and the Netherlands. It uses a participatory approach^53 54^ and is informed by the MRC framework for complex interventions.^55 56^

Phase I generates knowledge to inform intervention development through policy reviews, semi-structured interviews with stroke survivors and their family members, qualitative social network interviews,^57^ place-mapping techniques^58^ and cross-cultural adaptation and preliminary validation of CENS.^59^ Phase II applies a co-design process to develop and specify the intervention, including its core components, intended functions and principles for local adaptation. Phase III evaluates the intervention in a pragmatic pilot feasibility study, focusing on feasibility, acceptability, fidelity and implementation processes.

Intervention development in phases I and II will be reported in line with GUIDED,^60^ and the final intervention will be specified using the TIDieR framework.^61^ While participants from earlier phases may be invited to contribute to subsequent phases, each phase will generate analytically independent datasets. The project is not designed as a longitudinal cohort study, and participation across phases is not required.

At the time of manuscript revision, the project is in the early implementation stage. Start-up activities were undertaken in late 2024. During 2025, the consortium finalised study materials, initiated recruitment, commenced phase I data collection (including the policy review) and conducted a small number of initial interviews. The phase III pilot feasibility study is planned to commence no earlier than 2027. See online supplemental file 1 for data collection templates.

#### Target population and settings

The target population for EQL consists of individuals who have experienced stroke and are currently undergoing rehabilitation or recovery in a home-based setting in Sweden, the Netherlands and Latvia. This population includes adults aged 65 and older who have completed initial hospital-based acute treatment and are transitioning to rehabilitation at home with assistance from their family members or healthcare providers. Key characteristics of members of this population include the following:

- Individuals aged 65+ years have suffered stroke and who currently experience mild to moderate degrees of physical, cognitive or emotional impairments. These individuals should be undergoing ongoing rehabilitation or support in terms of activities of daily living to enhance their functional independence.
- These individuals should be in non-institutional, home-based settings and have access to basic medical support and caregiving (whether such support is provided formally or informally).

## Phase I: knowledge generation

### Design

Phase I uses a multimethod approach, including (1) policy document review, (2) semi-structured interviews with people and place mapping and (3) cross-cultural adaptation of CENS.

### Policy document review

We will review policy documents from Sweden, the Netherlands and Latvia to identify and describe how self-management support is framed within stroke rehabilitation. We will use the Practical Reviews in Self-Management Support Framework (PRISMS)^62^ to extract and map key components for self-management, such as patient education, shared decision-making, goal setting, action planning and problem-solving and to examine whether and how documents address social support, social networks and environmental factors. The review will be used to identify gaps and opportunities for improvement across systems.

### Interviews and place mapping

Semi-structured interviews will be conducted with three participant groups in each country: individuals with stroke, their family members and professionals involved in poststroke rehabilitation. The interviews will explore how different geographical contexts and social networks support or constrain rehabilitation and self-management, including perceived barriers to and enablers of the self-management strategies used by stroke survivors.

### Participants and recruitment

We aim to recruit approximately 10 stroke survivors, 10 family members and 5 professionals in each country (n=75) using purposive sampling^63^ to ensure variation relevant to the study aim. Table 1 summarises the inclusion criteria for participants in each interview set.

**Table 1:** Overview of study participants, recruitment and data collection methods

Aims and methods	Phase I *Policy study*	Phase I *Interview study*	Phase I *CENS study*	Phase II *Intervention study*	Phase III *Pilot study*
Aim	To analyse and document self-management strategies within healthcare systems in Sweden, Latvia and the Netherlands.	To investigate the role of social networks and environments in shaping recovery trajectories.	To validate cross-cultural measurement tools for assessing social network efficacy.	To develop an intervention for supported self-management intervention focusing on network efficacy.	To explore the relationship between physical and social environments, social networks, and self-management strategies in a pilot study.
Sample size	N/A	n=25 per country (10 stroke survivors, 10 family members, 5 professionals).	8–12 experts (CVI panel) + 6–8 stroke survivors (cognitive interviews) per country.	Approximately 15 participants per country (mixed stakeholder workshops).	20–40 stroke survivors + up to 20 family members.*
Participants	N/A	Individuals with stroke, family members and professionals.	A panel of stroke survivors, practitioners and researchers with experience working with stroke rehabilitation.	Individuals with stroke, family members and professionals.	Individuals with stroke, family members and professionals.
Inclusion criteria		Individuals with stroke: 65+ who have had a mild to moderate stroke. Be in non-institutional, home-based settings.	Individuals with stroke: 65+ who have had a mild to moderate stroke. Be in non-institutional, home-based settings.	Individuals with stroke: 65+ who have had a mild to moderate stroke. Be in non-institutional, home-based settings. Family members: Identified by stroke survivor. Professionals: Experience in stroke rehabilitation Working in home-based/community-based care	Stroke survivors aged 65+ years with mild to moderate impairments, living in non-institutional home-based settings and receiving rehabilitation or support. Family members identified by participating stroke survivors. Professionals involved in delivery of the intervention at the pilot site.
Recruitment strategy		Collaboration with healthcare sites and stroke organisations. Stroke survivors will be asked to identify a support person in their network.	Collaboration with healthcare sites and stroke organisations. Stroke survivors will be asked to identify a support person in their network.	Collaboration with healthcare sites and stroke organisations. Stroke survivors will be asked to identify a support person in their network.	Collaboration with healthcare sites and stroke organisations. Stroke survivors will be asked to identify a support person in their network.
Data collection method	Review of policy documents from Sweden, the Netherlands and Latvia.	Semi-structured interviews. Place-mapping Social network mapping.	CENS. CVI. Cognitive interviews.	Sequential and systematic co-design approach. Workshops in three countries.	Pre and post. Outcome measures: Self-Efficacy. Assessment. EQ-5D-5L. CENS. Process evaluation according to MRC. Interviews.
Analysis	The PRISMS framework will identify gaps in existing policies for potential improvement. The analysis will focus on how self-management support is incorporated into existing healthcare policies for stroke rehabilitation.	Reflexive thematic analysis.	CVI ratings will be used to calculate item-CVI (I-CVI) and the scale-CVI (S-CVI/CVI-Ave).	Material from the workshops will be critically reviewed and evaluated to generate an intervention guide.	Qualitative content analysis.

* The mix and sample size of professionals will vary according to the size of each site.

CENS, Collective Efficacy of Networks Scale; CVI, content validity index; EQ-5D-5, is a patient‑reported outcome measure developed by the EuroQol Group to describe and value a person’s current health status.; MRC, Medical Research Council; N/A, not available; PRISMS, Practical Reviews in Self-Management Support Framework.

Recruitment will occur through collaborating healthcare sites, stroke organisations and reference groups. Potentially eligible individuals will receive study information either face-to-face during a clinic visit or by post and will be invited to provide consent to be contacted for follow-up. Stroke survivors may nominate a family member who will be invited separately. Professionals will be recruited through partner sites and professional networks, with snowball sampling as needed.

### Data collection procedure

The interviews will be conducted by trained research staff via phone, video call or face-to-face at a time and place that is convenient for the participants. Once consent has been obtained, participants will complete a brief key demographic form. Interview topic guides will be used flexibly to elicit experiences of the transition home, everyday activities and self-management, and the role of social relationships and meaningful places (eg, local services, green spaces, relatives’ homes). We will also explore experiences of ‘lost’ people or places following stroke, including inaccessible environments and changes in relationships.^25^ Interviews will be adapted as needed to support participants with communication impairments, including the use of supported communication strategies and flexible pacing.

### People–place mapping

During the interview, we will use a so-called people–place map developed by Meijering and colleagues^58^ and add a ‘social’ component. Place mapping is a participatory visualisation technique that helps visualise the places and people that are important for someone as well as the links among these factors.

### Analysis

Data from each participant’s interviews will be analysed separately using reflexive thematic analysis.^64 65^ A small subset of the data will be double coded by two researchers in each country to refine the initial coding framework. Subsequent coding will be conducted using the agreed framework. Regular cross-country discussions will support analytic consistency and deepen interpretation.

### Translation and adopting the CENS

The translation and validation of the CENS will be conducted in all three countries. This task will involve several stages. Following permission from the original developers, two researchers will translate the scale into each language, and a native English speaker will back-translate. Discrepancies will be reviewed to produce a country-specific version for testing. Item relevance and clarity will be assessed using a content validity index (CVI) approach^66^ with an expert panel (8–12 stroke survivors, practitioners and researchers) and cognitive interviews with 6–8 stroke survivors in each country. CVI will be calculated at item and scale levels (I-CVI; S-CVI/Ave). Cognitive interviews will be transcribed verbatim and analysed using content analysis^67^ to identify comprehension issues and inform final refinements.

### Participants and recruitment

A panel of 8–12 stroke survivors, practitioners and researchers with experience working in the field of stroke rehabilitation will be asked to rate the relevance of each item and to comment on the translated scale. To obtain deeper insights into this topic, 6–8 stroke survivors will be interviewed based on a cognitive interview method. Participants in the panel and cognitive interviews will be recruited from each country via established reference groups (including stroke survivors, family members and professionals in stroke care and rehabilitation), stroke patient organisations and the research group’s network of researchers working in the field of strokes.

## Phase II: co-design and intervention specification

### Design

Phase II uses a participatory co-design approach, structured by the double diamond design process^68^ to translate phase I findings into intervention prototypes and to specify core components and functions of a network-based supported self-management intervention.

### Participants and recruitment

Participants will include stroke survivors, family members and healthcare professionals from the participating countries. We aim to include approximately 15 individuals in each country in the workshops. Recruitment will follow phase I pathways through collaborating sites, stroke organisations and reference groups. Participants in phase I will be invited to register their interest in participating in phase II but participation across sites is not required. Thus, additional participants will be recruited as needed to ensure adequate representation.

### Co-design process

Workshops will be facilitated by members of the research team and conducted onsite or in a hybrid format. Each workshop will last approximately 3 hours and will use structured activities to (1) prioritise needs and design requirements based on phase I findings, (2) generate and refine prototype components, (3) articulate hypothesised mechanisms of change and (4) identify what should be fixed versus adaptable across contexts. Across workshops, we will specify preliminary core components and their intended functions and develop principles for local adaptation while maintaining fidelity to core functions.^69 70^

We acknowledge that some individuals with stroke may experience speech, language or communication impairments (eg, aphasia). To support inclusive participation, workshops and related activities will be adapted as needed, including the use of supported communication techniques, simplified and visual materials, flexible pacing and the option to involve a trusted support person when appropriate.

### Outputs and analysis

Workshop materials (eg, notes, artefacts and agreed prototypes) will be collated and critically reviewed by the research team in collaboration with stakeholders. The primary output will be an intervention manual that specifies the purpose, content, delivery processes and adaptation principles of the NBI. We will also develop an initial logic model describing core components, intended functions and hypothesised pathways to outcomes, linking intervention processes to underpinning theoretical assumptions.

The finalised intervention will be specified using the TIDieR checklist^61^ framework to enable transparent description and replication.

## Phase III: pragmatic pilot feasibility evaluation and implementation learning

### Design

Phase III translates the intervention into practice and evaluates feasibility and acceptability. We will conduct a pragmatic pilot feasibility pilot study using a pre–post mixed-methods design with assessments at baseline (T0), postintervention (T1) and 8 weeks after completion (T2). The primary aim is to assess feasibility (recruitment, retention, fidelity, acceptability and data completeness) and to refine study procedures and outcome measurement.

### Setting

The pilot will be conducted at one or more sites within one consortium country, selected based on readiness and capacity to deliver the intervention in routine services. If feasible, additional pilot sites may be included. Site characteristics and contextual factors will be described to support interpretation.

### Participants and recruitment

We aim to recruit approximately 20–40 older stroke survivors living at home and up to 20 family members, consistent with recommendations for pilot feasibility studies.^71^ The number and mix of professionals involved will depend on site size and service configuration. Recruitment will occur via participating services and collaborating organisations using procedures described in phase I.

### Procedure

The pilot study will follow a structured three-phase process that aims to assess the feasibility and potential impact of the newly developed intervention.

#### Preintervention

Baseline measures and interviews will be conducted with people with stroke, their family members and professionals to capture support and self-management practices and contextual considerations.

#### The intervention phase

The intervention will be delivered in collaboration with the local team. EQL investigators will facilitate three co-learning workshops that last 3 hours with the healthcare professionals at the selected site. Subsequent follow-up seminars will be held to support integration into routine practice. A manual will guide delivery, and monthly outreach meetings with the local team will document fidelity, adaptations and implementation challenges.

#### Post-test phase

Postintervention and 8-week follow-up assessments will capture feasibility outcomes and candidate measures; follow-up interviews will explore participant and staff experiences, perceived usefulness and integration into practice.

### Outcome measures

**Feasibility outcomes** such as recruitment, intervention delivered and received; fidelity to core components and documented adaptations; acceptability to stroke survivors/family members and professionals; and completeness of outcome data at each time point. Measurement performance such as self-efficacy and quality of life will include health-related quality of life (EQ-5D-5L)^72^ and network efficacy (CENS^59^), alongside interview-based exploration of perceived self-management confidence. These data will be used to assess completion rates, variability and suitability of measures for future evaluation.

#### Self-efficacy assessment

Based on semi-structured interviews, we will explore patients’ confidence in their ability to manage their recovery following a stroke. These interviews will focus on key areas such as symptom management, daily activities and emotional/social challenges, and a flexible guide will be employed to capture individual experiences. Sample questions include “How confident do you feel in your ability to manage your health independently?” “What challenges do you encounter during your daily activities?” “How do you seek and use social support during your recovery?”

#### EQ-5D-5L

This standardised instrument features two parts and measures health-related quality of life.^72^ The first part of the instrument assesses five dimensions, that is, mobility, self-care, usual activities, pain/discomfort and anxiety/depression, which are scored on a five-level scale. The second part features a visual analogue scale that asks respondents to rate their overall health on a scale ranging from 0 (worst imaginable) to 100 (best imaginable). This combined approach provides a detailed health profile and elicits participants’ overall health perceptions. The scale has been used at the global level to assess quality of life after stroke, thus making it suitable for efforts to evaluate the quality of life of stroke survivors.^72^

#### Collective Efficacy of Networks Scale

This tool measures how individuals perceive their support network’s capacity to help them manage challenges.^59^ The scale features 13 items that are divided into the dimensions of structural support (which refers to the size and composition of the network) and perceived support (which refers to the quality and reliability of support); these items are scored on a Likert scale ranging from ‘Not at all’ to ‘Very best’. Validation studies have confirmed that the CENS is a reliable measure of the impact of social support on health outcomes.^59^

### Process evaluation

An embedded process evaluation will be conducted to examine contextual factors, implementation processes and mechanisms of change, guided by the MRC framework for process evaluations of complex interventions.^51^ Data sources will include interviews with professionals and with stroke survivors/family members, and documentation from outreach meetings capturing fidelity and adaptations.

### Analysis

Qualitative data will be analysed using qualitative content analysis,^67^ structured by the process evaluation domains (implementation, context and mechanisms). Quantitative data will be summarised descriptively (e.g. recruitment/retention, completion rates and distribution/variability of candidate measures) to inform refinement and planning of future evaluation. Table 1 provides a summary of the outcomes, measures/approaches and analyses corresponding to the study objectives. To evaluate the outcomes of the intervention, we will use validated instruments and interviews.

## Ethics and dissemination

The project has received ethical approval by ethics committees in each participating country: Sweden, the Swedish Ethical Review Authority: reg. no. 2025-00083-01 (registrator@etikprovning.se); Latvia, the Rīgas Stradiņa Universitāte Research Ethical Committee: reg.no. 2-PĒK-4/426/2025 (27 February 2025), 2-PĒK-4/567/2025 (27 March 2025); the Netherlands, the Research Ethics Committee of the Faculty of Spatial Sciences, University of Groningen: reg. no. 2025-07 (28 May 2025). The phase III pilot feasibility study will be prospectively registered in a public trial registry prior to participant recruitment. Findings will be disseminated through academic publications, stakeholder workshops and collaboration with stroke organisations to support translation into practice.

## Implications

EQL-stroke aligns with European priorities for life after stroke and will generate empirical data on the experiences of stroke survivors, their family members and professionals across three healthcare contexts. The project will provide practical recommendations for strengthening supported self-management after hospital discharge, including how social networks and everyday environmental context can be mobilised to support motivation, confidence and participation in meaningful activities.

For health services, EQL-stroke will produce a clearly specified intervention (with adaptation principles) and implementation learning from a pragmatic pilot feasibility evaluation, informing the development of home-based and community-based rehabilitation pathways. The multicountry collaboration provides an opportunity to identify context-dependent barriers and facilitators and to refine approaches that are feasible in routine practice.

The study’s contribution lies in integrating social networks, collective efficacy and everyday environmental context within a single supported self-management intervention for home-based stroke recovery, and in examining feasibility and implementation in diverse settings. Findings will inform future evaluation and potential scale-up across contexts.

## Supplementary material

10.1136/bmjopen-2025-110976 online supplemental file 1
