# Supplementary material for: Enhancing Quality of Life for Individuals with Stroke (EQL): a study protocol for co-creating a social support and context-informed intervention to improve self-management, health and well-being in older adults recovering at home
Source: BMJ Open. 2026 Apr 24;16(4):e110976. doi: 10.1136/bmjopen-2025-110976 (PMC13110533; doi:10.1136/bmjopen-2025-110976)
Supplement: online supplemental file 1 [file bmjopen-16-4-s001.docx]

**Interview and people–place mapping guides (English summary)**

**Template 1: Stroke survivor interview + people–place mapping**

**Purpose:** Explore self-management after stroke at home, focusing on social networks and meaningful places.

**Key domains:**

- Daily life after stroke; main challenges (physical, cognitive, emotional) and self-management strategies.
- Social network: who provides support (informal and formal); type of support (practical, emotional, informational); frequency and mode of contact (in-person/digital); changes in relationships after stroke.
- Places: important everyday places (home, local services, green spaces, family and friends’ homes); how access and mobility shapes participation; barriers and facilitators in the physical environment.
- “Lost” people, places, activities after stroke; what would enable re-engagement.
- Reflection on the mapping exercise and interview experience.

**Mapping procedure:** Participant co-creates a three-ring map placing key people closer, further from the centre based on importance; places are added and linked to people, activities; barriers, supports are noted and discussed.

**Template 2: Family member, friend interview + mapping**

**Purpose:** Understand support dynamics and sustainability for relatives/friends and the survivor.

**Key domains:**

- Network changes after stroke (roles, responsibilities, activity patterns).
- Who supports the survivor and the relative; which supports help and which are strained; desired changes in proximity/support.
- Important places and people for well-being and coping (e.g., associations, meeting places, healthcare, nature, faith communities, digital forums).
- Participation: what helps vs hinders social participation and everyday routines; how barriers are managed.

**Mapping procedure:** Three-ring map of people/groups/services relevant to survivors and relatives; places and activities are added.

**Template 3: Professional interview**

**Purpose:** Explore how professionals identify and work with patients’ social networks and contextual factors.

**Key domains:**

- How professionals learn about the patient’s social network (sources: patient or relative conversations, documentation).
- Perceived role of social networks in health and well-being; examples of helpful and insufficient support.
- Contact with relatives (frequency, purpose); perceived needs for network-informed care.
- How greater knowledge of social networks and place/context could improve rehabilitation and follow-up.

**Templates 4–5: CENS content validity and cognitive interviews**

**Purpose:** Support cross-cultural adaptation of CENS; assess item relevance, comprehensibility and usability.

**CVI panel:** Participants rate each item for relevance, clarity and ease of responding; free-text comments are collected.

**Cognitive interviews:** “Think-aloud” and probing questions explore interpretation of items, reasoning behind responses, and suggested rewording.
