# Supplementary material for: Enhancing Quality of Life for Individuals with Stroke (EQL): a study protocol for co-creating a social support and context-informed intervention to improve self-management, health and well-being in older adults recovering at home
Source: BMJ Open. 2026 Apr 24;16(4):e110976. doi: 10.1136/bmjopen-2025-110976 (PMC13110533; doi:10.1136/bmjopen-2025-110976)
Supplement: Supplementary file 2 [file bmjopen-2025-110976.reviewer_comments.pdf]

PEER REVIEW HISTORY

BMJ Open publishes all reviews undertaken for accepted manuscripts. Reviewers are asked to complete a checklist review form and are provided with free text boxes to elaborate on their assessment. These free text comments are reproduced below.

ARTICLE DETAILS

Title (Provisional)

Enhancing Quality of Life for Individuals with Stroke (EQL): A study protocol for co-creating a social support and context-informed intervention to improve self-management, health and well-being in older adults recovering at home

Authors

Kylén, Maya; Falk Johansson, Marcus; McCarthy, Linnea; Meijering, Louise; Tomsone, Signe; Tistad, Malin; Elf, Marie

---

VERSION 1 - REVIEW

**Reviewer**	**1**
**Name**	**Yoshihiro, Shodai**
**Affiliation**	**Hiroshima University Hospital, Department of pharmaceutical services**
**Date**	**07-Nov-2025**
**COI**	**None**

This manuscript appears to be a project proposal that combines at least three separate studies. It includes components of information gathering, intervention development, and evaluation of the developed intervention. However, descriptions of the three studies are insufficient, making it difficult to understand the novelty of the overall research. As a result, each study design is inadequately described and does not appear to follow the corresponding reporting guidelines such as STROBE, CONSORT, or TIDieR. Therefore, the appropriateness of each study design remains questionable.

The relationship between the novelty of the project and previous studies is also unclear from the background section. The authors need to explicitly identify the existing treatment gaps and clarify what kinds of interventions have been examined to date.

Each study design should be clearly delineated, and a separate protocol should be prepared for each phase. The project is planned to be conducted over five years, beginning in 2024. Considering the current time frame, part of the project may already have been completed.

This reviewer suggests that only Phase 3 be submitted as a protocol paper. The methods section could then summarize the development process of the intervention in Phases 1 and 2, followed by a detailed description of the finalized intervention. At present, it is difficult to determine how novel the developed intervention is compared with previously reported ones. Describing the intervention in detail according to the TIDieR checklist would help readers better understand its content and potential contribution. In addition, descriptions of the follow-up period are lacking.

**Reviewer**	**2**
**Name**	**Leslie, Paula**
**Affiliation**	**University of Pittsburgh**
**Date**	**12-Dec-2025**
**COI**	**None**

This is an is an extremely important and timely topic in the field of supporting older people post stroke, their families and the healthcare systems. I am very impressed with the intention of multi-centre and healthcare systems with varying resources which in turn will produce more generalisable outcomes. Care and thought has clearly gone into the design of this work. The points below are to support the researchers in optimising the robustness and applicability of the study, or to be reflected on for subsequent publications.

Overall: I appreciate the description of people with mild to moderate varying impairments post stroke. Given the importance of co-production and interview techniques etc, the impact of speech/language/communication impairments on the research should be clearly acknowledged. This is currently missing from the proposal.

Given the interest in recruiting subsequent cohorts from previous phases, how will the team manage attrition due to death, increasing illness, refusal to consent etc? And/or should subsequent cohorts be excluded so data are independent?

Page 11, line 51: dates need review as the project can't start until 2026 earliest.

Page 16, line 53: is [46] the correct reference?

Throughout: avoid utilis/ze and use “use” etc in line with clear writing guidance.

Requires information on trial registration.

VERSION 1 - AUTHOR RESPONSE

Reviewer 1	
This manuscript appears to be a project proposal that combines at least three separate studies. It includes components of information	Thank you for this important comment. We agree that the submitted version could be read as a single project proposal combining multiple

gathering, intervention development, and evaluation of the developed intervention. However, descriptions of the three studies are insufficient, making it difficult to understand the novelty of the overall research. As a result, each study design is inadequately described and does not appear to follow the corresponding reporting guidelines such as STROBE, CONSORT, or TIDieR. Therefore, the appropriateness of each study design remains questionable.	activities, and that the three phases were not described with sufficient clarity for readers to judge the novelty and methodological appropriateness of each part.<br><br>In response, we have substantially revised the manuscript. The overall multi-phase structure is pre-specified in the accepted, funded programme (THCS/EU and national funders), and we have therefore not changed the underlying design. However, we have restructured the manuscript to present the study as a multi-phase master protocol, with each phase described as a clearly delineated sub-study with its own aims, design, participants, procedures, outcomes and analysis plan.<br><br>We have also strengthened alignment with reporting guidance by using a phase-appropriate approach. Phase 1 (policy review and qualitative interviews with people–place mapping, including CENS adaptation) is now described using an appropriate qualitative/observational protocol structure.<br><br>Phase 2 (co-design and intervention development) is presented as intervention development work, and the intervention specification is strengthened through a TIDieR-informed description.<br><br>Phase 3 is now explicitly framed as a pragmatic pilot feasibility study using a single-arm pre–post mixed-methods design with an 8-week follow-up, with feasibility, acceptability, fidelity and process evaluation as the primary focus (rather than definitive effectiveness estimation).<br><br>We also revised the background to clarify the treatment/support gap and the novelty of the overall study, including the rationale for integrating social networks and environmental context into supported self-management after stroke.<br><br>We appreciate the point of using reporting frameworks. Rather than applying a single reporting framework across all phases, we have clarified the methodological logic and reporting approach for each phase. Because this

	manuscript is a multi-phase protocol (not a report of completed trial/observational results), we use phase-appropriate protocol structure and guidance: intervention development guidance (GUIDED), TIDieR for specification of the finalised intervention, and a clearly defined pilot feasibility design with MRC-informed process evaluation for Phase 3.<br><br>With respect to the suggestion to submit only Phase 3, we understand the rationale and have addressed this concern by making the phases methodologically distinct and transparent within one manuscript. However, in this case, reporting only the pilot feasibility study would not adequately represent the pre-specified THCS-funded design or provide sufficient context for understanding how the intervention was developed, specified and adapted for feasibility testing. We therefore retained the multi-phase protocol format, while substantially improving the clarity and evaluability of each phase.
The relationship between the novelty of the project and previous studies is also unclear from the background section. The authors need to explicitly identify the existing treatment gaps and clarify what kinds of interventions have been examined to date.	Thank you for this comment. We have substantially revised the Background to make the treatment/support gap more explicit and to improve the positioning of EQL-stroke relative to previous work. Specifically, we now clarify that while prior post-stroke self-management interventions have largely focused on education, behaviour change and clinically delivered support, fewer interventions have explicitly integrated social networks and everyday environmental contexts as core components of supported self-management in home-based recovery.<br><br>We also clarify how EQL-stroke extends previous work through a co-designed, network-based intervention with cross-country adaptation and feasibility testing in routine care.
Each study design should be clearly delineated, and a separate protocol should be prepared for each phase.	Thank you for this suggestion. We agree that the study designs needed clearer delineation. In the revised manuscript, it is now clearer that EQL-stroke is a multi-phase master protocol, with three clearly defined sub-studies contributing to the overall development and feasibility evaluation of the network-based intervention.
The project is planned to be conducted over five years, beginning in 2024. Considering the current time frame, part of the project may	Thank you for noting this. We have corrected the timeline to reflect the current project schedule, including that the Phase 3 pilot feasibility study is planned to commence no

already have been completed.	earlier than 2027. We have also added a brief project status at submission statement to transparently distinguish completed start-up/preparatory activities (and limited early Phase 1 activities) from the remaining prospective data collection.
This reviewer suggests that only Phase 3 be submitted as a protocol paper. The methods section could then summarize the development process of the intervention in Phases 1 and 2, followed by a detailed description of the finalized intervention. At present, it is difficult to determine how novel the developed intervention is compared with previously reported ones. Describing the intervention in detail according to the TIDieR checklist would help readers better understand its content and potential contribution. In addition, descriptions of the follow-up period are lacking.	Thank you for this thoughtful and constructive suggestion. We appreciate the rationale for submitting only Phase 3 as a protocol paper and summarising Phases 1–2 as developmental work. We agree that, in the original version, the intervention development process and the description of the finalised intervention were not presented clearly enough for readers to assess novelty and methodological coherence.<br><br>In the revised manuscript, we have retained the multi-phase protocol format because EQL-stroke is a pre-specified THCS-funded project in which Phases 1–3 are sequentially linked and jointly inform the intervention and its feasibility evaluation. However, we have substantially revised the manuscript to address your concerns within this format.<br><br>Specifically, we have:<br>(i) clarified the multi-phase structure and delineated each phase as a distinct sub-study with phase-specific aims, design, participants, procedures and analysis;<br><br>(ii) strengthened the background to more explicitly identify the treatment/support gap and to position EQL-stroke in relation to previously studied self-management interventions;<br>(iii) strengthened the intervention description and specification (including a TIDieR-informed description of the planned/finalised intervention and its core components/functions, with adaptation principles); and<br><br>(iv) clarified Phase 3 as a pragmatic pilot feasibility study (single-arm pre–post mixed-methods) with an explicit 8-week follow-up and an embedded process evaluation, with feasibility/acceptability/fidelity as the primary focus rather than definitive effectiveness.<br><br>We hope these revisions make the intervention's rationale, development pathway and potential

	contribution clearer, while preserving the logic of the funded multi-phase protocol.
Reviewer 2.	
This is an is an extremely important and timely topic in the field of supporting older people post stroke, their families and the healthcare systems. I am very impressed with the intention of multi-centre and healthcare systems with varying resources which in turn will produce more generalisable outcomes. Care and thought has clearly gone into the design of this work.<br><br>The points below are to support the researchers in optimising the robustness and applicability of the study, or to be reflected on for subsequent publications.	We thank the reviewer for the encouraging comments. We also agree that this is a timely area where stronger, more applicable evidence is needed across different healthcare contexts. In the revised manuscript, we have strengthened the rationale by linking EQL-stroke to current European priorities.<br>For example, the Stroke Alliance for Europe (SAFE) and the European Stroke Organisation's Stroke Action Plan for Europe has recently been updated (mid-term review), with an explicit emphasis on improving "life after stroke" support, including supported self-management methods, involvement of family and friends, and attention to the person's wider context and relationships. We have added this to the background to clarify how our focus on network efficacy, social support and place/context aligns with these updated European recommendations and addresses identified gaps in long-term rehabilitation and follow-up.
Overall: I appreciate the description of people with mild to moderate varying impairments post stroke. Given the importance of co-production and interview techniques etc, the impact of speech/language/communication impairments on the research should be clearly acknowledged. This is currently missing from the proposal.	Thank you for this important observation. We agree that speech, language, and communication impairments are highly relevant in stroke research, particularly within participatory and co-design approaches. We have now explicitly addressed this in both Phase 1 (interviews) and Phase 2 (co-design) sections. The manuscript now clarifies that interviews and workshops will be adapted as needed using supported communication techniques, simplified and visual materials, flexible pacing, and the optional involvement of a trusted support person.
Given the interest in recruiting subsequent cohorts from previous phases, how will the team manage attrition due to death, increasing illness, refusal to consent etc? And/or should subsequent cohorts be excluded so data are independent?	Thank you for this important methodological point. We have clarified in the manuscript that the project is not designed as a longitudinal cohort study. Although participants from earlier phases may be invited to contribute to later phases, participation across phases is not required. Each phase generates analytically independent datasets, and analyses are conducted separately. This design reflects both pragmatic considerations and the anticipated challenges of maintaining a stable cohort over a

	multi-year, multi-country project involving older adults recovering from stroke.
Page 11, line 51: dates need review as the project can't start until 2026 earliest.<br><br>Page 16, line 53: is [46] the correct reference?<br>Throughout: avoid utilis/ze and use "use" etc in line with clear writing guidance.	**Page 11, line 51: dates need review as the project can't start until 2026 earliest.**<br>Thank you for noting this. We have reviewed and updated the project timeline to reflect the current schedule. The manuscript now clarifies that the pilot feasibility study will commence no earlier than 2027, following completion of the preceding development phases.<br><br>**Page 16, line 53: is [46] the correct reference?**<br>We have carefully reviewed reference 46 and confirmed that it correctly refers to the person–environment–occupation (PEO) model.<br><br>**Throughout: avoid utilis/ze and use "use" etc in line with clear writing guidance.**<br>Thank you for this helpful suggestion. We have revised the manuscript throughout to improve clarity and consistency of language, replacing terms such as "utilise/utilize" with "use" and simplifying phrasing in line with clear writing guidance.
Requires information on trial registration.	To be registered prior to recruitment (Phase 3 pilot feasibility study).
